# Placement of dual capsular tension rings for the combined management of traumatic cyclodialysis cleft and zonular dialysis

**DOI:** 10.1186/s40662-020-00219-x

**Published:** 2020-11-23

**Authors:** Jiahui Chen, Lina Lan, Yating Tang, Yi Lu, Yongxiang Jiang

**Affiliations:** 1 Eye Institute and Department of Ophthalmology, Eye Ear Nose and Throat Hospital, Fudan University, 83 Fenyang Rd, Shanghai, 200031 China; 2 NHC Key Laboratory of Myopia (Fudan University), Key Laboratory of Myopia, Chinese Academy of Medical Sciences, Shanghai, 200031 China; 3 Shanghai Key Laboratory of Visual Impairment and Restoration, Shanghai, 200031 China

**Keywords:** Capsular tension ring, Cyclodialysis cleft, Zonular dialysis, Combined surgery

## Abstract

**Background:**

To evaluate the efficacy and safety of placing dual capsular tension rings for the combined management of traumatic cyclodialysis cleft and zonular dialysis.

**Methods:**

A modified capsular tension ring was inserted into the ciliary sulcus and a capsular tension ring or modified capsular tension ring was inserted into the capsular bag in 20 eyes in 20 consecutive patients showing signs of ocular hypotony and ectopia lentis. Outcome measures included intraocular pressure, best-corrected visual acuity, and postoperative complications.

**Results:**

Dual capsular tension ring placement was performed in 20 patients with a mean age of 48.7 years. The cyclodialysis cleft extended over 2.9 clock hours (range 0.5–6.5). The modified capsular tension ring was successfully inserted into the ciliary sulcus and a capsular tension ring or modified capsular tension ring in the capsular bag in all eyes. At the last follow-up, the cyclodialysis cleft was closed in 16/20 (80.0%) eyes. The intraocular lens was stable in all patients postoperatively. Best-corrected visual acuity, in terms of the logarithm of the minimal angle of resolution, improved from 1.3 ± 0.8 before surgery to 0.4 ± 0.3 after surgery (*P* < 0.001). Intraocular pressure increased significantly from 10.6 ± 3.2 mmHg before surgery to 13.0 ± 4.8 mmHg after surgery (*P* = 0.040). Postoperative complications included a painful reversible intraocular pressure spike in four patients (20.0%). Logistic regression revealed no significant factors associated with successful cleft closure and a stable final intraocular pressure of ≥ 10 mmHg.

**Conclusions:**

The placement of two capsular tension rings into the ciliary sulcus and the capsular bag is a safe, successful procedure combined for repairing a traumatic cyclodialysis cleft and managing zonular dialysis.

## Background

Cyclodialysis clefts form due to incorrect insertion of the meridional ciliary fibers in the scleral spur, creating a secondary pathway that allows aqueous humor to drain into the suprachoroidal space [1–3]. A cyclodialysis cleft combined with zonular dialysis is a rare disorder that usually occurs following blunt ocular trauma, and usually leads to persistent hypotony, cataract, and ectopia lentis. It is often difficult to diagnose and treat this traumatic complication due to its rarity and subtle clinical signs [1]. Timely detection and accurate management to restore the apposition of the detached ciliary body and the dislocated lens are vital to ensure postoperative visual recovery.

Various non-surgical and surgical methods to close the cyclodialysis cleft and center the dislocated lens have been proposed [4–13]. Gupta et al. [11] reported a case of cataract, zonular dialysis, cyclodialysis, and iridodialysis following blunt trauma that was successfully treated in a single procedure by placing two capsular tension rings (CTRs). We previously performed phacoemulsification combined with an internal tamponade by insertion of a modified capsular tension ring (MCTR) into the ciliary sulcus to close cyclodialysis clefts and treat cataracts [12, 13]. Appropriate positioning of a polymethyl methacrylate CTR or Cionni MCTR in the sulcus apposing the detached ciliary body against the sclera provides stable endocyclotamponade and endocyclopexy (in-out suture to aid fixation), which promotes cleft closure [8, 9, 13, 14]. Similarly, implantation of an MCTR, an adjacent zonular apparatus, achieved the best possible outcomes in terms of long-term stability and centering of the intraocular lens (IOL) in patients with ectopia lentis following continuing refinement of surgical techniques [15–17].

Surgical interventions are usually essential for traumatic cyclodialysis cleft, which is often accompanied by other ocular injuries. Performing multiple surgical procedures in a single operation can avoid the burden associated with multiple procedures, general anesthesia, and postoperative morbidity, and can make the procedure more cost-effective for the patient and hospital. In this study, we describe the efficacy and safety of inserting an MCTR into the ciliary sulcus and a CTR or MCTR into the capsule for traumatic cyclodialysis cleft and zonular dialysis in a single procedure, in a series of consecutive patients with cyclodialysis cleft and zonular dialysis.

## Methods

### Ethics approval

The Institutional Review Board of the Eye and ENT Hospital of Fudan University, Shanghai, China, approved this study (No.2013021). All procedures were conducted following the tenets of the Declaration of Helsinki. Written informed consent was obtained from all patients.

### Patient eligibility

Between January 2017 and December 2019 inclusive, consecutive patients attending the ophthalmology clinic at the Eye and ENT Hospital of Fudan University, Shanghai, China, who fulfilled the eligibility criteria were enrolled in this prospective case-series study. Patients with a traumatic ocular injury resulting in cyclodialysis cleft and zonular dialysis and who underwent combined insertion of an MCTR into the ciliary sulcus and a CTR or MCTR into the capsular bag were eligible. Exclusion criteria were concomitant vitreoretinal surgery, spontaneous closure, or closure by direct cyclopexy. Cyclodialysis cleft and zonular dialysis were confirmed by ultrasound biomicroscopy (UBM).

### Surgical technique

**Fig. 1:**
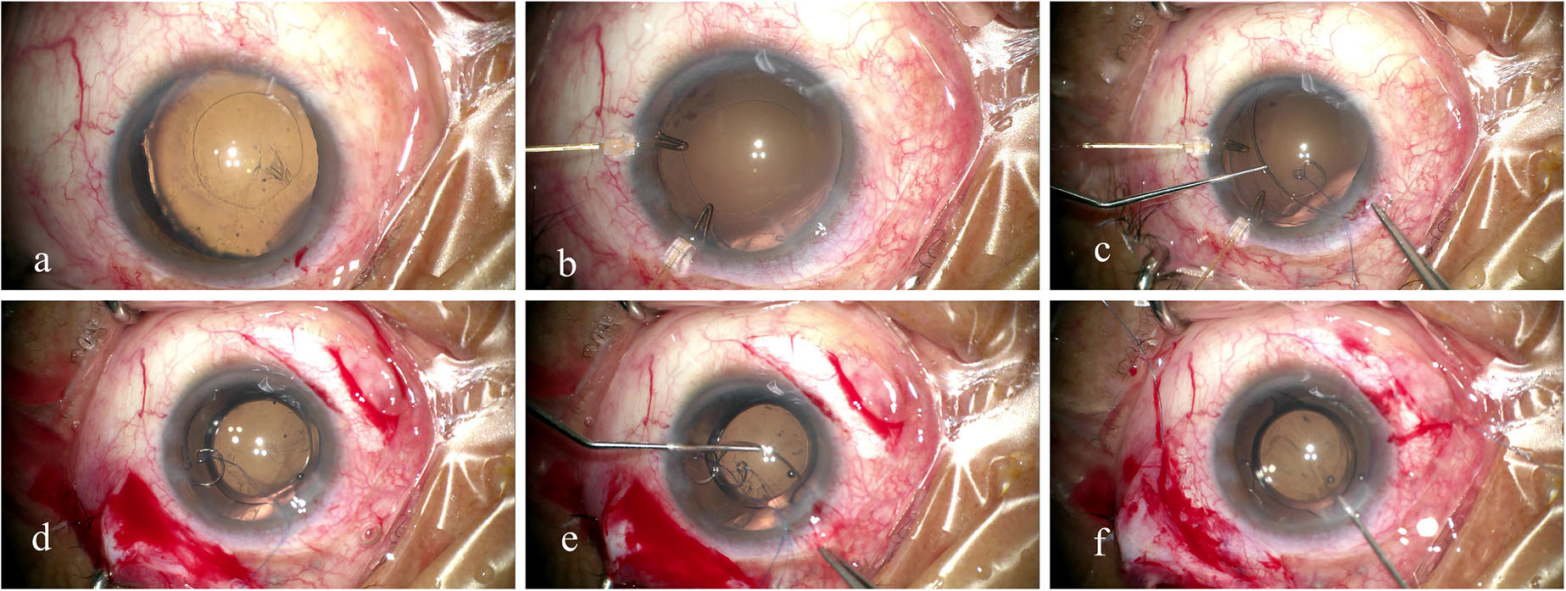
Surgical microscopic images showing insertion of a combined Cionni-modified capsular tension ring (MCTR) into the capsular bag and an MCTR into the sulcus for repairing cyclodialysis cleft and zonular dialysis. **a** Ectopia lentis with a round capsulorhexis in a patient of cyclodialysis cleft. **b** Fixation of two capsule hooks for capsular support. **c** Insertion of an MCTR with one eyelet into the capsular bag. **d** Implantation of an intraocular lens into the capsular bag. **e** Insertion of an MCTR with two eyelets into the ciliary sulcus. **f** Separation of the sutures that fix the eyelets of both MCTRs

A superior clear corneal tunnel incision and paracentesis were made under retrobulbar anesthesia or general anesthesia. An ophthalmic viscosurgical device (OVD) was injected intracamerally to enable anterior continuous curvilinear capsulorhexis, the fixation of capsule hooks, and hydrodissection. Following standard stop and chop or tilt and tumble phacoemulsification technique and cortical aspiration, the viscocohesive OVD was then re-injected into the capsular bag. A CTR (Morcher Type 14C, Type 14A or Type 14, GmbH, Germany) or an MCTR (Morcher Type 1 L or Type 2C, GmbH, Germany) with one eyelet preset with 9–0 polypropylene aligned along the area of zonular dialysis was implanted into the capsular bag to support the zonules, after which a foldable IOL was inserted into the capsule. In some eyes, the cortices were aspirated after inserting the CTR or MCTR into the capsular bag. After dealing with the zonular dialysis, a 13-mm Morcher Type 2 L CTR (Morcher GmbH, Germany), an MCTR with two eyelets preset with 10–0 polypropylene was then positioned into the ciliary sulcus, and the end of the prolene suture was tied to the sclera 1 mm posterior to the surgical limbus. The position of one eyelet was pushed towards the maximum height of the cleft, which resulted in the reattachment of the detached ciliary body to the scleral spur by direct mechanical tamponade. The surgery was concluded by aspirating the OVD and closing the corneal wound (Fig. 1).

### Outcome measures

Preoperative data included demographic features, trauma history, duration of trauma, best-corrected visual acuity (BCVA), intraocular pressure (IOP), anterior chamber depth (ACD), number and size of the cyclodialysis clefts, ectopia lentis, and coexisting ophthalmic pathologies. Slit-lamp examination, B-scan ultrasonography, optical coherence tomography (OCT5000, Carl Zeiss Meditec, Inc., Dublin, CA, USA), and UBM (MD-300 L, MEDA, Tianjin, China) were performed in all patients. Measures of success included an increase in IOP to ≥ 10 mmHg, confirmation of cleft closure on UBM, and confirmation of IOL centration under slit-lamp examination. Postoperative data included the duration of follow-up, the maximum IOP, IOP stability, and the proportion of eyes with successful cleft closure.

### Statistical analysis

Statistical analyses were performed using SPSS version 23.0 (IBM Corp., Armonk, NY, USA). Descriptive statistics are presented as the mean ± standard deviation and range, where appropriate. Student’s paired *t*-test was used to compare preoperative and postoperative measurements within the whole group. The Student’s *t*-test was applied for comparisons between the patients with and without retinopathy. Logistic regression was used to determine which factors were associated with successful cleft closure or IOP recovery using age, the time between trauma and surgery, preoperative parameters, and cleft size as possible factors. The results of two-sided tests were considered statistically significant at *P* < 0.05.

## Results

A total of 20 patients (20 eyes) were enrolled and underwent dual insertion of CTRs to repair a cyclodialysis cleft and correct ectopia lentis. An MCTR with two eyelets was successfully inserted into the ciliary sulcus in all patients, a CTR was inserted into the capsular bag in 13 patients and an MCTR with one eyelet was inserted in seven patients. The mean ± SD age of the patients at the time of surgery was 48.7 ± 10.7 years (range, 31–74 years) and 75% were male. The affected eye was the right eye in 12 patients (60%). The procedure was performed at a mean of 7.9 months after ocular trauma (range, 15 days to 36 months), excluding one patient in whom the duration was unclear from the medical records. The baseline clinical features of the patients can be found in Table 1.

**Table 1:** Demographic parameters and ocular characteristics of patients with cyclodialysis cleft and zonular dialysis

No.	Sex	Age (years)	Eye	Cause	Number of Cleft	Extension (Clock Hours)	Duration of Cleft (months)	BCVA (LogMAR)	IOP (mmHg)	ACD (mm)	CTR or MCTR in the capsule	No. of surgery	IOP spike	Associated pathology
1	M	48	L	Hard stick	1	2.5	14	2.0	14.8	2.63	CTR	2	Yes	traumatic cataract, choroidal detachment
2	M	47	R	Crabstick	1	2.0	12	2.9	7.4	2.53	MCTR	1	No	traumatic cataract, hypotonic maculopathy, choroidal detachment
3	F	55	R	Firewood	1	1.5	24	0.9	8.2	1.74	CTR	1	No	traumatic cataract, choroidal detachment
4	F	69	R	Traffic accident	1	3.0	36	1.4	14.5	2.17	MCTR	1	No	traumatic cataract, choroidal detachment, angle recession
5	M	47	R	Fist	1	2.5	2	1.1	16.8	1.74	CTR	1	No	traumatic cataract, choroidal detachment, vitreous hernia
6	M	31	R	Billboard	1	3.5	16	1.0	7.9	2.09	CTR	2	Yes	traumatic cataract, epiretinal membrane, choroidal detachment, angle closure
7	M	36	L	Plank	1	2.0	1	1.0	8.0	2.53	MCTR	1	No	traumatic cataract, iridodialysis, choroidal detachment
8	M	44	L	Fist	1	2.0	1	0.4	10.0	2.79	MCTR	1	No	traumatic cataract, hypotonic maculopathy, choroidal detachment
9	F	47	R	Firecrackers	1	0.5	7	0.5	9.0	2.36	CTR	1	No	traumatic cataract, hypotonic maculopathy, choroidal detachment, iris bombe
10	F	74	R	Pick	1	2.5	12	0.9	13.7	1.38	MCTR	1	No	traumatic cataract, epiretinal membrane, angle closure
11	M	54	R	Hoe	1	4.0	1	0.7	14.9	2.33	MCTR	1	No	traumatic cataract, choroidal detachment, angle closure
12	M	46	L	Valve	1	2.5	12	1.3	8.8	2.20	CTR	1	No	traumatic cataract, hypotonic maculopathy, choroidal detachment, angle recession
13	F	49	R	Crabstick	1	4.5	0.5	1.0	6.0	1.47	CTR	1	No	traumatic cataract, choroidal detachment, hyphema
14	M	35	R	Grinding wheel	1	5.5	1	2.2	12.0	1.97	CTR	2	No	traumatic cataract, iridodialysis, choroidal detachment
15	M	55	L	Elbow	1	3.0	5	1.5	7.0	2.20	CTR	1	No	traumatic cataract, choroidal detachment, macular edema
16	M	45	L	Tumble	1	1.0	/	0.4	10.0	1.71	CTR	1	No	traumatic cataract, macular edema, choroidal detachment
17	M	46	L	Elastic band	1	3.0	1	0.2	11.0	2.05	CTR	1	No	choroidal detachment, angle closure
18	M	35	R	Polished sheet	1	6.5	1	1.9	7.0	2.07	CTR	1	Yes	traumatic cataract, iridodialysis, macular edema
19	M	55	L	Traffic accident	1	3.5	2	0.8	11.2	2.03	CTR	1	Yes	traumatic cataract, choroidal detachment, macular edema, iris bombe
20	M	56	R	unclear	1	3	2	3.0	13.5	1.97	MCTR	1	No	traumatic cataract, hypotonic maculopathy, choroidal detachment, angle closure, vitreous hernia

*BCVA* best-corrected visual acuity, *logMAR* logarithm of the minimal angle of resolution, *IOP* intraocular pressure, *ACD* anterior chamber depth, *CTR* capsular tension ring, *MCTR* modified capsular tension ring

**Fig. 2:**
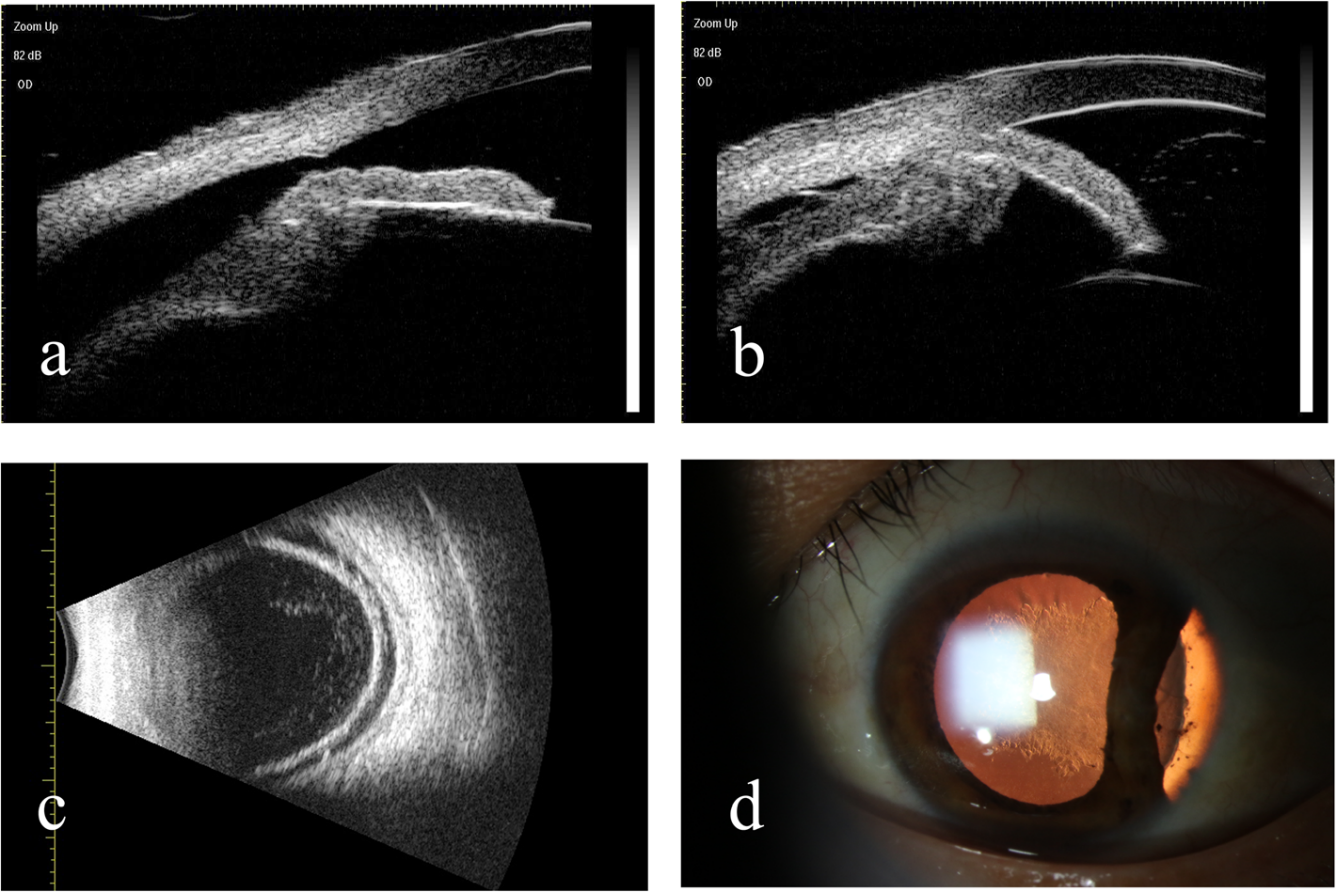
Preoperative clinical images in representative cases of cyclodialysis cleft and zonular dialysis. **a** & **b** Preoperative ultrasound biomicroscopy image showing cyclodialysis cleft and zonular dialysis for the same patients. **c** Preoperative B-scan ultrasonographic image showing the detachment of choroid. **d** Preoperative slit-lamp biomicroscopic photograph showing temporal iridodialysis, zonular disruption with a subluxated lens, and a posterior subcapsular cataract

All patients had a single cyclodialysis cleft (Fig. 2a & b). The extent of the cyclodialysis cleft was 2.9 ± 1.4 clock hours (range 0.5–6.5), and most of the patients (90%) had a large cleft (≥ 1.5 clock hour). The location of the cleft varied and the cleft affected one quadrant (3 clock hours) in 14 patients, one to two quadrants (3 to 6 clock hours) in five patients, and three quadrants (> 6 clock hours) in one patient.

Significant ocular complications included traumatic cataract in 19/20 eyes (95.0%), choroidal detachment in 18 eyes (90.0%, Fig. 2c), anterior chamber angle closure in five eyes (25%), hypotonic maculopathy in five eyes (25.0%), macular edema in four eyes (20.0%), iridodialysis in three eyes (15.0%, Fig. 2d), vitreous hernia in two eyes (10.0%), angle recession in two eyes (10.0%), and epiretinal membrane in two eyes (10.0%). Three eyes had previously undergone surgical procedures, including external direct cyclopexy for cleft repair in two patients (#1 and #6) and corneal laceration suturing in one patient (#14). Vitreoretinal surgery was not performed in any of our patients. The pre- and post-operative BCVA and IOP were not significantly different between patients with and without retinopathy (all *P* > 0.05, data not shown).

The preoperative BCVA, in logarithms of the minimal angle of resolution (logMAR), ranged from 0.2 to 3.0 (mean ± SD 1.3 ± 0.8) and preoperative IOP ranged from 6.0 to 16.8 mmHg (mean ± SD 10.6 ± 3.2 mmHg; 1 mmHg = 0.133 kPa). Postoperatively, BCVA (logMAR) improved to 0.4 ± 0.3 (*P* < 0.001) and IOP increased significantly to 13.0 ± 4.8 mmHg (*P* = 0.040). Postoperatively, the IOP returned to normal (≥ 10 mmHg) in 15 eyes (75.0%) at different times after the procedure. Four patients (20.0%) experienced a postoperative IOP spike several days after surgery, of which two required antiglaucoma drugs to control their temporary IOP elevation. The postoperative IOP spikes were reversible, and none of the patients had a persistent elevated IOP and none required antiglaucoma surgery.

**Fig. 3:**
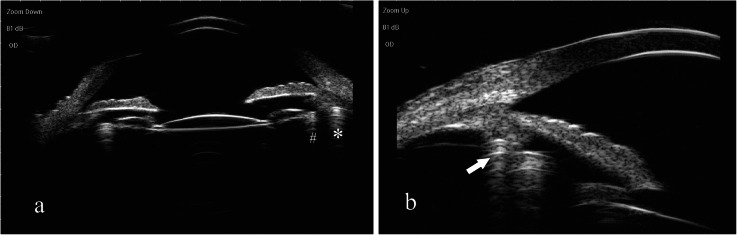
Postoperative ultrasound biomicroscopy images of dual capsular tension rings insertion in the ciliary sulcus and the capsule. **a** & **b** Ultrasound biomicroscopy showing a double indentation sign (arrow) after inserting a Cionni-modified capsular tension ring in the ciliary sulcus and a capsular tension ring in the capsular bag. The two arc-shaped strong echoes with multiple reflections on the picture indicate the Cionni-modified capsular tension ring (*) and the capsular tension ring (#) (The two pictures below are taken from the same patient)

No intraoperative complications such as the shallow anterior chamber, capsular hook slipping, vitreous prolapse, iris injury, anterior capsular tear, and posterior capsular rupture, occurred in any patients. All of the patients were followed up at a mean ± SD of 2.3 ± 1.9 months after the procedure. At the last follow-up, the cyclodialysis cleft was successfully closed in 16/20 (80.0%) eyes, indicating a favorable anatomic prognosis. IOL was stable in all patients (20 eyes, 100.0%) postoperatively. The confirmation of cleft closure was shown on UBM and a double indentation sign of two arc-shaped strong echoes with multiple reflections was presented after reattaching the ciliary body to the scleral spur by a Cionni MCTR and inserting a CTR into the capsular bag (Fig. 3).

Logistic regression revealed that successful cleft closure was not associated with age (*P* = 0.310), duration of hypotony (*P* = 0.340), cleft size (*P* = 0.176), preoperative BCVA (*P* = 0.662), or preoperative IOP (*P* = 0.790). Furthermore, a final IOP of ≥ 10 mmHg was not significantly associated with age (*P* = 0.616), duration of hypotony (*P* = 0.430), cleft size (*P* = 0.501), preoperative IOP (*P* = 0.147), postoperative IOP spike (*P* = 0.458), and preoperative ocular complications (*P* = 0.387 for choroidal detachment; *P* = 0.572 for iridodialysis).

## Discussion

The repair of traumatic dialyses is vital to restore eye anatomy and achieve optimal visual outcomes. In eyes with a small cyclodialysis cleft (< 1.5 clock hours), conservative therapy offered good prognosis following medical and argon laser photocoagulation [18–20]. However, surgical procedures are inevitable for larger cyclodialysis clefts, especially in eyes with coexisting cataract and ectopia lentis, and a single procedure may be preferred over multiple surgical procedures [13].

Owing to the variable manifestations of cyclodialysis cleft and coexisting complications of ocular trauma, ophthalmic surgeons need evidence-based guidance and must select the most appropriate intervention that can effectively close the cleft and treat the complications [2]. Although the surgical procedure described here may be lengthy, this is offset by the short postoperative rehabilitation period. To date, we have performed combined surgical procedures in 20 patients with traumatic cyclodialysis cleft, zonular dialysis, and other associated pathologies, none of whom have experienced severe complications such as intraocular hemorrhage, anterior chamber shallowing, capsular hook slipping, vitreous prolapse, iatrogenic iridodialysis, anterior capsular tear, or posterior capsular rupture. BCVA improved and IOP increased significantly postoperatively. This approach using dual CTRs was also minimally invasive and technically straightforward.

A cyclodialysis cleft forms following detachment of the longitudinal ciliary muscle fibers from the scleral spur and incorrect insertion of the ciliary body from the origin of the lens zonular fibers may result in an illusion of zonular weakness and lens subluxation [1, 13, 21]. Once the ciliary body is mechanically reapposed to the scleral spur, there is no need to insert a CTR or MCTR into the capsular bag because the zonules tighten and the capsule centers. Zonular dialyses with different circumferences of involvement are manifested by zonular weakness and displacement of the crystalline lens, and they should be carefully evaluated before surgery [22]. Zonular dialysis, which is frequent in ocular trauma, can lead to decentration and tilt of the capsule–IOL complex postoperatively, so an MCTR should be inserted into the capsular bag to ensure stable long-term support [23]. In our study, 13 of the 20 patients underwent in-the-bag CTR insertion and seven patients underwent in-the-bag insertion of an MCTR with one eyelet, maintaining perfect IOL centration.

A cyclodialysis cleft creates an abnormal pathway for the drainage of the aqueous humor into the suprachoroidal space, leading to hypotony and choroidal detachment. However, some patients in the present study showed an IOP of > 10 mmHg in the presence of cyclodialysis cleft and choroidal detachment. The ocular concomitant pathology, such as the shallow anterior chamber, anterior segment inflammation, vitreous prolapse, peripheral anterior synechiae, anterior chamber angle closure, and anterior displacement of the lens might lower the outflow of aqueous humor and increase IOP.

Internal tamponade together with implantation of a CTR and IOL into the ciliary sulcus has also been proposed for repairing a cyclodialysis cleft [7, 9, 14, 24]. After rotating one of the IOL haptics to face the site of the cyclodialysis cleft, the IOL forces the ciliary body to the sclera spur, thereby closing the cyclodialysis cleft [7, 24]. However, the potential risk of ciliary body erosion, hemorrhage, pain, and inflammation from the compressive effects of the stiff IOL haptics should not be ignored [3]. In eyes with a large cleft, the IOL haptic would not provide adequate support by direct apposition of the ciliary body to the sclera spur. Inserting a CTR into the capsular bag can provide stability for the capsule–IOL complex, but the CTR does not exert a direct tamponade force to reattach the detached ciliary body to the ciliary sulcus because the capsule may limit the extent of CTR expansion. To restore ocular anatomy, an in-the-bag IOL is a more reasonable approach to avoid postoperative refractive surprise, despite a reduction in axial length due to hypotony, and hence achieve optimal visual recovery [9].

As a mean diameter of the ciliary sulcus is 12.0 to 12.5 mm in nearly emmetropic adult eyes, a non-compressed Morcher Type 2 L MCTR with a diameter of 13.0 mm is suitable for side-to-side placement and presumably effective internal tamponade in the ciliary sulcus. Using the optimal MCTR size, the force was exerted not only by the ring on the ciliary sulcus to provide internal tamponade, but also ab-interno suturing of the eyelet of the MCTR for reattachment of the ciliary body.

In cases with cyclodialysis cleft combined with vitreous hemorrhage, vitrectomy, endophotocoagulation, and gas/silicone oil endotamponade treatment are irreplaceable techniques [25]. In the absence of preoperative vitreoretinal severe complications, patients with hypotonic maculopathy may achieve good visual recovery after cleft repair and IOP normalization. In this study, patients suffered from cyclodialysis cleft and other ocular complications including traumatic cataract, ectopia lentis, choroidal detachment, refractive error, hypotonic maculopathy, macular edema, and epiretinal membrane, all of which usually affect vision substantially. Postoperative visual acuity seems to be mainly determined by the presence of macular scars and retinal detachment rather than by the extension of the dialysis or the presence of concomitant pathology since we found that pre- and post-operative BCVA did not differ significantly between patients with and without retinopathy.

There were four patients with unsuccessful cleft closure at the last follow-up. Two patients with sustained 4- and 4.5-clock hour clefts had incomplete cleft closure but their IOP stabilized at 10 mmHg. It is speculated that the production and outflow rate of aqueous humor might reach an equilibrium in both of these patients as traumatic synechia or iridial inflammation increased the IOP and some small clefts decreased the IOP. Therefore, they did not undergo further treatments, and a long-term follow-up was scheduled. One case with a 3-clock hour cleft had multiple complications, including traumatic cataract, ectopia lentis, macular edema, choroidal detachment, and angle recession. B-scan ultrasonography examination showed a slight detachment of the choroid. Further UBM examination should be done to confirm the existence of a small cleft during the follow-up. This patient may need argon laser treatment to improve the IOP to more than 10 mmHg. The fourth case was a young male with a 6-clock hour cyclodialysis cleft. Closure of the most severe clefts was confirmed, but small clefts remained, and they required long recovery time and laser photocoagulation treatment.

Some key aspects of our procedure warrant attention. First, for patients with the insertion of two MCTRs, it is vital to determine the most severe position of the dislocated lens and the maximum height of the cleft preoperatively, to ensure the sutures that fix both MCTRs are passed around the interlamellar sclera and the two eyelets of the dual MCTRs should be staggered. Second, if miosis occurs during MCTR insertion into the ciliary sulcus, it is helpful to switch from capsular retractors to iris hooks. Third, when inserting an MCTR with two eyelets into the ciliary sulcus, the suture placed on the side of the cleft should be pulled tightly, whereas the opposite suture should be fixed gently to avoid warping the MCTR against the iris. Finally, it is not necessary to completely aspirate the peripheral OVD because a high postoperative IOP facilitates cleft closure.

We appreciate there are some limitations to estimating the surgical outcomes of dual CTRs insertion for the treatment of cyclodialysis and zonular dialysis in a small cohort, which is due to the relative rarity of this pathology. The results of our efforts to develop a safe and minimally invasive method for the treatment of ocular traumatic dialyses seem to be favorable. However, larger controlled studies are needed to verify the results. In this short-term study, we did not perform comparisons with other surgical methods, such as inserting an IOL into the ciliary sulcus, although we did observe a favorable success rate following dual CTRs insertion. Our study provides an alternative option for ophthalmologists to treat ocular dialyses.

## Conclusions

A traumatic cyclodialysis cleft complicated with zonular dialysis is an uncommon complication that requires successful repair to ensure anatomic correction and improvement in visual function. Delaying treatment might lead to a permanent visual deterioration and severe sequelae of ocular trauma. Prompt intervention is therefore recommended in complex ocular dialyses, and the repair of cyclodialysis clefts and the correction of zonular dialysis can be managed in a combined procedure, involving the insertion of dual CTRs into the ciliary sulcus and the capsular bag.

## Data Availability

The datasets generated and/or analyzed during the present study are not publicly available (obtained from Eye and Ear, Nose, and Throat Hospital, Fudan University, Shanghai repository), but are available from the corresponding author upon reasonable request.

## Abbreviations

ACD: Anterior chamber depth
BCVA: Best-corrected visual acuity
CTR: Capsular tension ring
IOL: Intraocular lens
IOP: Intraocular pressure
logMAR: Logarithms of the minimal angle of resolution
MCTR: Modified capsular tension ring
OVD: Viscosurgical device
UBM: Ultrasound biomicroscopy
